# Adult granulosa cell tumor of the testis masquerading as hydrocele

**DOI:** 10.1590/S1677-5538.IBJU.2014.0187

**Published:** 2015

**Authors:** Archana George Vallonthaiel, Aanchal Kakkar, Animesh Singh, Prem N Dogra, Ruma Ray

**Affiliations:** 1 Department of Pathology, All India Institute of Medical Sciences, New Delhi, India; 2 Departments of Urology, All India Institute of Medical Sciences, New Delhi, India

**Keywords:** Granulosa Cell Tumor, Sex Cord-Gonadal Stromal Tumors, Testis, Immunohistochemistry, Neoplasms, Germ Cell and Embryonal

## Abstract

Adult testicular granulosa cell tumor is a rare, potentially malignant sex cord-stromal tumor, of which 30 cases have been described to date. We report the case of a 43-year-old male who complained of a left testicular swelling. Scrotal ultrasound showed a cystic lesion, suggestive of hydrocele. However, due to a clinical suspicion of a solid-cystic neoplasm, a high inguinal orchidectomy was performed, which, on pathological examination, was diagnosed as adult granulosa cell tumor.

Adult testicular granulosa cell tumors have aggressive behaviour as compared to their ovarian counterparts. They may rarely be predominantly cystic and present as hydrocele. Lymph node and distant metastases have been reported in few cases. Role of MIB-1 labelling index in prognostication is not well defined. Therefore, their recognition and documentation of their behaviour is important from a diagnostic, prognostic and therapeutic point of view.

## INTRODUCTION

While granulosa cell tumor represents the most common sex cord-stromal tumor arising in the ovary (1), and juvenile testicular granulosa cell tumor (TGCT) is the commonest sex cord-stromal tumor seen in male children, adult TGCT remains an enigmatic entity. Due to its rarity, not much is known about its natural course; however, literature suggests that adult TGCTs are slow-growing neoplasms with potential for lymph node metastasis, even many years after initial diagnosis. We report a case of this rare tumor which was predominantly cystic, causing a diagnostic dilemma clinically, and ultimately diagnosed on histopathology.

### Case Report

**Figure 1:**
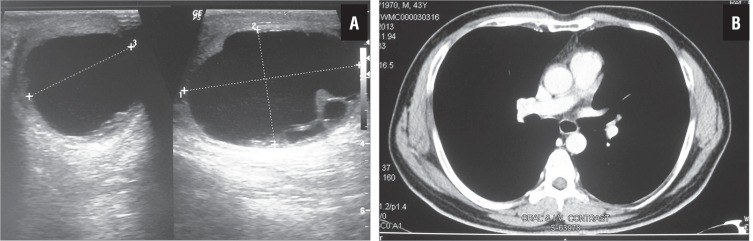
Scrotal ultrasound showing a cystic lesion with nodular soft tissue shadows at the periphery (a). CT abdomen one year post-surgery shows no lymphadenopathy (b).

This 43 years old male, under follow-up in the Urology clinic for stone disease, complained of painless, progressively increasing left testicular swelling for two months. On physical examination, vitals were stable. No abdominal distension or mass was noted. Peripheral lymphadenopathy was absent. Left testicular enlargement was identified, caused by a cystic scrotal swelling. Scrotal ultrasound (Figure-1a) showed an anechoic cystic lesion measuring 5.5cm × 3.4cm, with nodular soft tissue shadows at the periphery and only a thin rim of testicular tissue. Based on ultrasonography, differential diagnoses included intra-testicular cystic neoplasm and a cystic lesion compressing the testis. On investigation, routine haematological and biochemical parameters, as well as serum alpha-fetoprotein (AFP), lactate dehydrogenase and human chorionic gonadotropin levels were within normal limits. The patient was counselled for and submitted to a high-inguinal orchidectomy. Post-operative period was uneventful. The patient is doing well one year after surgery. CT abdomen revealed no retroperitoneal lymphadenopathy (Figure-1b).

### Pathological examination

**Figure 2:**
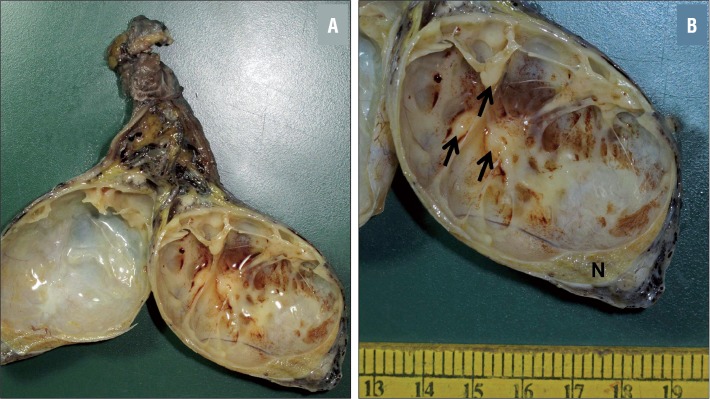
Orchidectomy specimen showing a solid cystic tumor (a); multiple cysts with few small nodules (arrows) are seen, along with a rim of normal testicular parenchyma (N) at the periphery (b).

Left high inguinal orchidectomy specimen comprised of testis measuring 8cm × 5.5cm × 4.5cm, with attached spermatic cord measuring 6cm. A predominantly cystic tumor (Figure-2a) measuring 6cm × 3.5cm × 2.5cm was identified, almost completely replacing the testis, with a thin rim of compressed normal testicular parenchyma at the lower pole. Cysts varied from 0.5cm to 5cm in diameter, were smooth-walled, and contained clear fluid. Few solid nodules, 0.4cm to 1cm in maximum dimension, were seen within the cyst walls (Figure-2b). The tumor did not appear to infiltrate the tunica albuginea. No areas of hemorrhage or necrosis were identified.

**Figure 3:**
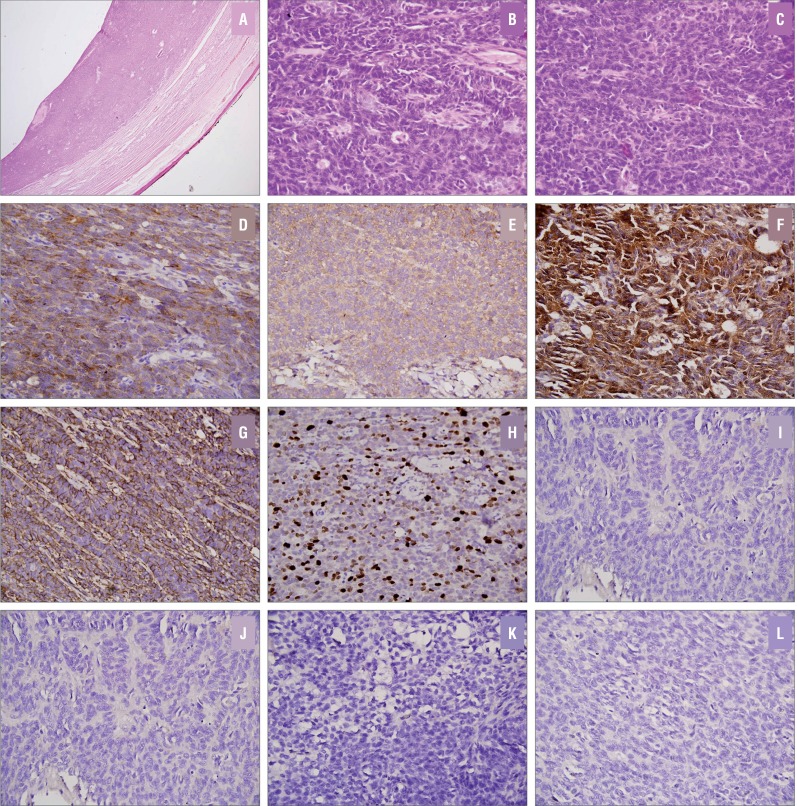
Photomicrographs showing solid areas of tumor along with compressed seminiferous tubules at the periphery (a; HE, x40); tumor cells were arranged in trabeculae and microfollicles, had scant cytoplasm and ovoid nuclei with grooves (b; HE, x400); frequent mitotic figures seen (c; HE, x400). Tumor cells were positive for inhibin (d), MIC2 (e), calretinin (f) and vimentin (g), MIB1-LI was high (h); EMA (i), AFP (j), synaptophysin (k) and CD117 (l) were negative (IHC, x400)

On microscopic examination (Figure-3), the tumor was well-circumscribed, unencapsulated, and was composed of monomorphic cells arranged in sheets and trabeculae. Focally, microfollicular structures (Call-Exner bodies) were seen. Tumor cells had scant cytoplasm, ill-defined cytoplasmic borders, and ovoid medium-sized nuclei with fine chromatin and inconspicuous nucleoli. Longitudinal nuclear grooves were seen at places. Frequent mitotic figures (8–10/10 high power fields) were identified. No necrosis, lymphovascular invasion or pseudosarcomatous areas were present. On immunohistochemistry, tumor cells were diffusely immunopositive for vimentin, inhibin, MIC2, and calretinin. They were negative for pancytokeratin (CK), epithelial membrane antigen (EMA), leukocyte common antigen (LCA), AFP, placental alkaline phosphatise (PLAP), CD117 and synaptophysin. MIB-1 labelling index (LI) was high (18% in highest proliferating areas). Based on histomorphological and immunohistochemical features, a diagnosis of adult granulosa cell tumor of the testis was made. Section from resected end of spermatic cord was free of tumor.

## DISCUSSION

Adult testicular granulosa cell tumor (TGCT) is a rare sex cord-stromal neoplasm arising in the testes, of which thirty cases have been reported until now (2–15). Majority of patients present with testicular enlargement which is painless and of variable duration (3). Some may present with features of hormonal dysregulation, like gynaecomastia (4, 5). In some instances, however, this tumor may be detected incidentally, as in our case (6). The age range at presentation is wide, varying from 16 to 77 years (2).

Adult TGCTs occur as solid, circumscribed tumors, in contrast to the multicystic juvenile TGCTs. While it is not unusual to identify small cystic foci within adult TGCTs (1), predominantly cystic tumors are rarely encountered, contributing to lack of suspicion of this diagnosis preoperatively, as in the case under discussion. Only two cases have been previously documented as presenting with hydrocele on the same side of tumor (7, 8).

A variety of microscopic patterns may occur in adult TGCTs, making differential diagnosis from Sertoli-Leydig cell tumor, germ cell tumors, non-Hodgkin lymphoma and neuroendocrine tumors difficult. Microfollicular, macrofollicular, trabecular, and insular growth patterns are commonly observed (3). Typical Call-Exner bodies may or may not be present. These tumors are immunopositive for vimentin, inhibin, MIC2, calretinin, and smooth muscle actin (3), and are usually negative for CK, EMA, LCA, synaptophysin, chromogranin, PLAP, and AFP (9). Morphological features along with immunohistochemistry help in clinching the correct diagnosis (Table-1). A recent review by Rabban et al. succinctly summarizes the immunohistochemical differential diagnosis between sex cord stromal tumors and germ cell tumors using newer IHC markers (16). While PLAP is a fairly reliable marker for germ cell tumors like dysgerminoma, yolk sac tumor, choriocarcinoma, and embryonal carcinoma, SALL4 is a novel marker that has higher sensitivity for the above-mentioned tumors with the exception of choriocarcinoma. OCT4 is a transcription factor which is expressed in dysgerminoma and gonadoblastoma. Sex cord-stromal tumours including adult granulosa cell tumours are, however, negative for PLAP, OCT4 and SALL4. Glypican-3 is another new marker with high specificity for yolk sac tumors, and is not expressed in sex cord stromal tumors. Newer markers for the sex cord stromal family of tumors include SF-1 and FOXL2, which are highly specific and are not expressed by germ cell tumors.

**Table 1:** Immunohistochemical panel for differential diagnosis of adult testicular granulosa cell tumor from morphologically similar tumors.

Differential diagnosis	Vimentin	CK	EMA	Inhibin	Calretinin	MIC2	PLAP	CD117	SALL4 OCT4	Glypican-3	SF-1/FOXL2	AFP	LCA	Chromogranin/Synaptophysin
Adult TGCT	+	-/focal	-	+	+	+	-	-	-	-	+	-	-	-
Seminoma	+/-	-/+	-	-	-	-	+	+	+	-	-	-	-	-
Yolk sac tumor	-	+	-	-	-	-	+	-	+	+	-	+	-	-
Non-Hodgkin lymphoma	+	-	-	-	-	-	-	-	-	-	-	-	+	-
Sertoli-Leydig cell tumor	+	+	-/+	+	+	+	-	-	-	-	+	-	-	-
Neuroendocrine tumors	-	-	-	-	-	-	-	-	-	-		-	-	+

**CK =** cytokeratin; **EMA =** Epithelial membrane antigen; **PLAP =** Placental alkaline phosphatase; **AFP =** alpha feto protein; **LCA =** leukocyte common antigen

In contrast to their more common juvenile counterparts that have a benign course and are cured by simple orchidectomy, adult TGCTs are believed to be potentially malignant tumors, which behave more aggressively than their namesakes in the ovary (2, 3). Reports of metastases to lymph nodes as well as distant sites, even as late as ten years after initial surgery, have served to consolidate this opinion (10). Definitive criteria for malignancy are lacking, and identification of aggressive cases remains difficult. Few features have been proposed by Jimenez-Quintero et al. (10), including size greater than 7cm, lymphovascular invasion, necrosis, and hemorrhage, while Hanson et al. have suggested that tumors greater than 5cm may behave aggressively (3). However, it is not clear whether size of the entire tumor should be taken into consideration in tumors that are solid-cystic, or only the solid areas. The role of MIB-1 LI (proliferation marker) in predicting malignant behavior is not clear and needs to be ascertained. As our case showed high MIB-1 LI, the patient was followed-up closely, but has remained free of disease one year post-surgery.

In conclusion, adult TGCTs are potentially malignant tumors that may have unusual clinical presentation occasionally. Recognition of these rare neoplasms and documentation of their behaviour is important from diagnostic, prognostic and therapeutic points of view. As delayed lymph nodal and distant metastases have been reported, patients need to be followed-up over a prolonged period after orchidectomy. Utility of MIB-1 LI in identification of tumors that may behave aggressively needs further evaluation.

## References

[1] Sesterhenn A, Cheville J, Woodward PJ, Damjanov I, Jacobsen GK, Nistal M, Eble JE, Sauter G, Epstein JI, Sesterhenn IA (2004). Sex cord / gonadal stromal tumours. Pathology and Genetics of Tumours of the Urinary System and Male Genital Organs.

[2] Miliaras D, Anagnostou E, Moysides I (2013). Adult type granulosa cell tumor: a very rare case of sex-cord tumor of the testis with review of the literature. Case Rep Pathol.

[3] Hanson JA, Ambaye AB (2011). Adult testicular granulosa cell tumor: a review of the literature for clinicopathologic predictors of malignancy. Arch Pathol Lab Med.

[4] Matoska J, Ondrus D, Talerman A (1992). Malignant granulosa cell tumor of the testis associated with gynecomastia and long survival. Cancer.

[5] Laskowski J (1952). Feminizing tumours of the testis: General review with case report of granulosa cell tumour of the testis. Endokrynol Pol.

[6] Wang BY, Rabinowitz DS, Granato RC, Unger PD (2002). Gonadal tumor with granulosa cell tumor features in an adult testis. Ann Diagn Pathol.

[7] López JI (2007). Adult-type granulosa cell tumor of the testis. Report of a case. Tumori.

[8] Hisano M, Souza FM, Malheiros DM, Pompeo AC, Lucon AM (2006). Granulosa cell tumor of the adult testis: report of a case and review of the literature. Clinics (São Paulo).

[9] Hammerich KH, Hille S, Ayala GE, Wheeler TM, Engers R, Ackermann R (2008). Malignant advanced granulosa cell tumor of the adult testis: case report and review of the literature. Hum Pathol.

[10] Jimenez-Quintero LP, Ro JY, Zavala-Pompa A, Amin MB, Tetu B, Ordoñez NG (1993). Granulosa cell tumor of the adult testis: a clinicopathologic study of seven cases and a review of the literature. Hum Pathol.

[11] Song Z, Vaughn DJ, Bing Z (2011). Adult type granulosa cell tumor in adult testis: report of a case and review of the literature. Rare Tumors.

[12] Gupta A, Mathur SK, Reddy CP, Arora B (2008). Testicular granulosa cell tumor, adult type. Indian J Pathol Microbiol.

[13] Al-Bozom IA, El-Faqih SR, Hassan SH, El-Tiraifi AE, Talic RF (2000). Granulosa cell tumor of the adult type: a case report and review of the literature of a very rare testicular tumor. Arch Pathol Lab Med.

[14] Ditonno P, Lucarelli G, Battaglia M, Mancini V, Palazzo S, Trabucco S (2007). Testicular granulosa cell tumor of adult type: a new case and a review of the literature. Urol Oncol.

[15] Arzola J, Hutton RL, Baughman SM, Mora RV (2006). Adult-type testicular granulosa cell tumor: case report and review of the literature. Urology.

[16] Rabban JT, Zaloudek CJ (2013). A practical approach to immunohistochemical diagnosis of ovarian germ cell tumours and sex cord-stromal tumours. Histopathology.

